# Effect of the cardiac long non-coding RNA Charme depletion on the maturation and paracrine signaling of resident cardiac fibroblasts

**DOI:** 10.1038/s41419-026-08636-x

**Published:** 2026-04-15

**Authors:** Erica Floris, Claudia Cozzolino, Giulia Buonaiuto, Valeria Taliani, Antonella Bordin, Alessandro Palma, Vittorio Picchio, Xhulio Dhori, Francesco Nutile, Carmine Nicoletti, Giacomo Frati, Monica Ballarino, Isotta Chimenti, Francesca Pagano

**Affiliations:** 1 Department of Medical Surgical Sciences and Biotechnologies, Sapienza University of Rome, Latina, Italy; 2 Department of Biology and Biotechnologies “Charles Darwin”, Sapienza University of Rome, Rome, Italy; 3 EMBL, Genome Biology Unit, Heidelberg, Germany; 4 Department of AngioCardioNeurology, IRCCS Neuromed, Pozzilli, Italy; 5 CINECA, Super Computing Applications and Innovation Department, Rome, Italy; 6 DAHFMO-Unit of Histology and Medical Embryology, Sapienza University of Rome, Rome, Italy; 7 Maria Cecilia Hospital, GVM Care & Research, Cotignola, Italy; 8 Institute of Biochemistry and Cell Biology, National Council of Research (IBBC-CNR), Monterotondo, Italy; 9 Present Address: Department of Medical Surgical Sciences and Biotechnologies, Sapienza University of Rome, Latina, Italy

**Keywords:** Cell biology, Molecular biology

## Abstract

*Charme* is a murine long non-coding RNA necessary for the embryonic development of the heart. In vivo Charme knock-out causes prominent alterations of tissue structure, due to cardiac hyperplasia, and leads to the development of cardiac dysfunctions. Cardiac fibroblasts (CFs) play pivotal roles in both the development and homeostatic maintenance of the cardiac tissue, and the effect of Charme depletion on this cell compartment has not been addressed. This study investigated the phenotype and function of resident CFs isolated from Charme knockout (Charme^KO^) mice and revealed their impaired maturation and functionality. Charme^KO^ hearts show decreased levels of collagen I content in the extracellular matrix, associated with reduced extracellular matrix-related gene expression and matrix remodeling ability of CFs. Charme^KO^ CFs show impaired phenotypic conversion into myofibroblasts and reduced responsiveness to activation stimuli, accompanied by the retention of features proper of unactivated mesenchymal cells. Charme depletion also affects CF paracrine function, determining an impoverishment in cardioprotective cytokines in their secretome, with consequent reduced ability to mediate PI3K/Akt pathway activation in cardiomyocytes and to induce the angiogenic process in endothelial cells. Charme^KO^ CFs also proved to be less supportive of cardiomyocyte maturation in an in vitro model of cardiac differentiation, thus indicating a potential contribution to the impairment of cardiomyocyte maturation in vivo. Overall, the evidence collected suggests that Charme depletion in the heart impairs CF maturation capacity and ECM deposition function, which can contribute to the alterations observed in the Charme^KO^ mice. These findings pave the way to deeper investigations on the intercellular signaling occurring in the heart upon Charme ablation to identify how microenvironment homeostasis contributes to cardiomyocyte maturation and is potentially involved in cardiac diseases.

## Introduction

Long non-coding RNAs (lncRNAs) are a heterogeneous class of non-protein coding transcripts, playing pivotal roles in many biological processes, such as cell growth and differentiation, survival and apoptosis, tissue and organ development and function [1–3]. Many cardiac-specific lncRNAs have been identified to be involved in heart development, and their dysregulation has been associated with disease [4, 5].

*Charme* (Chromatin architect of muscle expression) is a murine lncRNA involved in the myogenic process [6], whose expression is restricted to skeletal and cardiac muscles [7–9], also presenting a conserved human orthologue, *HSCHARME* [10], with 45% sequence identity. In mice, the chromatin associated *pCharme* isoform controls the expression of myogenic genes [7, 8], many of which are altered in various familial cardiac diseases, including hypertrophic, dilated, and arrhythmogenic human cardiomyopathies [10, 11]. Ablation of nuclear *pCharme* determines alterations of the trabecular myocardium and reduced capillary endothelium density in mice during cardiac development, with a marked cardiac hyperplasia and thickening of the ventricular walls [9]. Charme^KO^ mice also develop cardiac dysfunction in late adult life, characterized by significant left ventricular dilation and reduced fractional shortening, up to overt dilated cardiomyopathy [8].

Alongside the parenchymal contractile cells, the cardiac tissue includes a variety of stromal cells, characterized by heterogeneity and plasticity, playing key roles in tissue development, homeostasis, and pathological conditions [12, 13]. Multiple subpopulations of cardiac fibroblasts (CFs) have been described with different functional states [14]. They support cardiomyocyte differentiation during development [15] and maintain tissue homeostasis throughout life by extracellular matrix (ECM) synthesis and remodeling, paracrine communication, and cell-to-cell interaction with the other cardiac cell types [16, 17]. Indeed, CFs influence cell survival and stress resistance, angiogenesis, and immune cell activation [18–21] in both physiological and pathological conditions [22]. The activation of the stromal compartment becomes particularly important after injury due to its key role in repair and scarring mechanisms [23].

In this study, we investigated the phenotype and function of resident CFs in murine Charme^KO^ hearts. We hypothesized that the altered cardiac structure observed in the Charme^KO^ hearts might be associated with a phenotypic alteration of the fibroblast cell compartment. Thus, we assessed the features of the cardiac tissue in adult Charme^KO^ mice, and identified impairment in both tissue ECM and phenotypic conversion of Charme^KO^ CFs into myofibroblasts. Charme^KO^ CFs showed indeed altered ability to synthesize and remodel the ECM, reduced functional activation and differentiation into myofibroblasts, and significant impairment of the paracrine action on other cell types, including CMs, overall accompanied by the retainment of hallmarks of un-activated CFs, such as self-renewal properties and motility.

## Results

### Charme^KO^ cardiac fibroblasts have reduced expression of extracellular matrix organization genes, associated with a decreased collagen I deposition

**Fig. 1:**
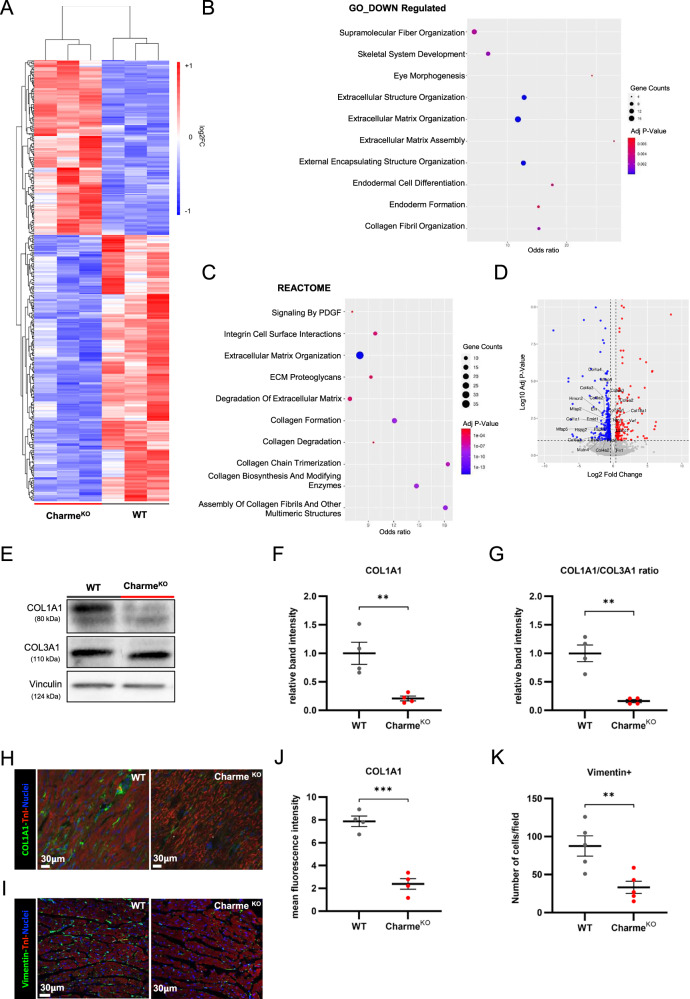
Charme^KO^ cardiac stroma has reduced expression of ECM synthesis and organization genes, reflected in cardiac tissue decreased collagen I content. **A** Heatmap showing the clustering of significant differentially expressed genes (DEGs) in native CFs isolated from Charme^KO^ versus WT murine hearts. **B** Bubble graph of the significantly enriched Gene Ontology terms retrieved by the GO analysis of down-regulated DEGs. Numerosity of each category, adjusted p value and odds ratio of the enrichment terms are reported. **C** Bubble plot showing the association of the DEGs with molecular pathways inferred using Reactome database. Numerosity of each category, adjusted p value and odds ratio of the enrichment terms are reported. **D** Volcano plot of the DEGs showing significantly up-regulated and down-regulated genes. Labels of genes belonging to the ECM categories retrieved in GO analysis are shown. **E** Representative western blot images showing the amount of collagen I and collagen III proteins. α/β-Tubulin was used as the loading control. **F** Densitometric analysis of the blots for collagen I, with the band intensity normalized over tubulin. **G** Collagen I to collagen III normalized band OD ratio. Quantification was made on four independent biological replicates. **H** Representative confocal microscopy images on heart sections from Charme^KO^ and WT mice after immunofluorescence staining for collagen I (green) and troponin I (red), counterstained for nuclei (blue). **I** Representative confocal microscopy images on heart sections from Charme^KO^ and WT mice after immunofluorescence staining for vimentin (green) and troponin I (red), counterstained for nuclei (blue) **J** Quantification of mean fluorescence intensity for collagen I immunostaining in Charme^KO^ heart sections. **K** Quantification of vimentin-positive cell number per field. For all dot plots, mean ± S.E.M. was indicated. N≥3; *p < 0.05; ***p < 0.001.

In Charme^KO^ mice, no evidence has been collected so far regarding the transcriptome of the non-cardiomyocyte population, particularly fibroblasts which constitute approximately 11% of all cardiac cells [24] and regulate the ECM composition and amount. Therefore, CFs were freshly isolated from adult (5-week-old) WT and Charme^KO^ hearts (Supplementary Fig. 1A), tested for the absence of hematopoietic (CD45) and vascular (CD309) markers, as well as for the presence of the mesenchymal/fibroblast markers CD90 and Sca1 (Supplementary Fig. 1B), and subjected to RNA-sequencing. The expression of *pCharme* was evaluated and confirmed to be very low, as expected [9] (Supplementary Fig. 1C). Moreover, transcriptomic analysis showed that the top expressed genes in the native CFs are related to ECM production and organization (Supplementary Fig. 1D). The comparison between Charme^KO^ and WT native CFs revealed a good separation between samples (Supplementary Fig. 1E, F) and 366 statistically significant differentially expressed genes (DEGs), with 145 genes up-regulated and 221 down-regulated in Charme^KO^ CFs compared to the WT counterpart (Supplementary Table 1). Hierarchical clustering of the statistically significant DEGs showed a clear distinction in the transcriptomic profile of Charme^KO^ CFs (Fig. 1A). Despite the function of Charme as chromatin architect, the observed effect on the transcriptome is unlikely to becaused by a direct structural remodeling of adult CFs, as the lncRNA is not expressed in this cell type. Instead, the effect is t possibly determined by impaired paracrine signaling of Charme^KO^ cardiomyocytes to CFs. Gene Ontology (GO) analysis of the significant DEGs evidenced the enrichment in categories related to ECM secretion and remodeling, and cardiac morphogenesis, for the downregulated (Fig. 1B) or upregulated genes (Supplementary Fig. 1G), respectively. Similarly, the analysis for enriched pathways highlighted terms related to the ECM and, more specifically, collagen synthesis and organization (Fig. 1C). Several genes responsible for the ECM deposition and composition were among the most abundantly expressed and significantly downregulated in Charme^KO^ cells (Fig. 1D, Supplementary Fig. 1H). Particularly, the genes encoding for collagen subunits were found downregulated (Supplementary Fig. 1I, J), and a significant reduction of collagen I was detected by western blot (WB) in the whole myocardial tissue of Charme^KO^ as compared to WT mice (Fig. 1E, F). Conversely, collagen III protein showed no change (Fig. 1E), yielding a consequent reduction of collagen I-to-collagen III (COL1A1/COL3A1) protein ratio (Fig. 1G). Both the reduced collagen I and stable collagen III content of the heart tissue were further confirmed by immunofluorescence staining on tissue sections from Charme^KO^ and WT mice (Fig. 1H, J; Supplementary Fig. 1K, L). The number of CFs, identified as vimentin-positive cells counted per field, was also reduced in the heart of adult Charme^KO^ mice (Fig. 1I, K). Overall, these data show that Charme absence leads to the alteration of the cardiac interstitium, characterized by reduced CF number and density, collagen I deposition, and a consequent imbalance of the collagen I-to-collagen III ratio.

### Charme^KO^ cardiac fibroblasts show impaired phenotypic conversion to myofibroblast

**Fig. 2:**
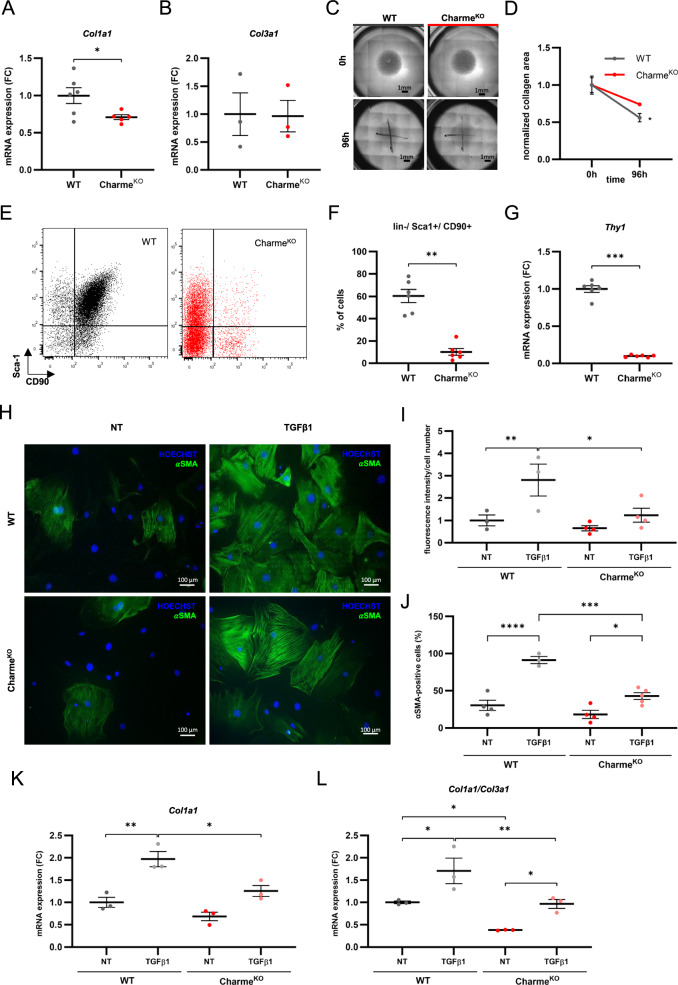
Charme^KO^ cardiac fibroblasts reveal impaired phenotypic conversion and are less responsive to TGF-β1-induced activation. **A**, **B** Dot plots showing *Col1a1* and *Col3a1* expression respectively, quantified by real-time qPCR expressed as fold change normalized on the WT samples. **C** Representative images and **D** normalized area quantification of a collagen matrix digestion assay up to 96 h of culture. **E** Representative dot plots for the gating strategy of flow cytometry analysis of CFs for CD90 and Sca1 quantification. **F** Dot plot showing the average percentage of lin-/Sca1+/CD90+ cells in Charme^KO^ vs WT CFs. **G** Dot plot showing *Thy1* gene expression quantified by real-time qPCR as fold change normalized on the WT samples. **H** Representative 10X magnification images of immunofluorescence staining on WT and Charme^KO^ CFs, either not treated (NT) or treated with TGF-β1 for 72 h (green: αSMA; blue: HOECHST). **I** Dot plot showing mean fluorescence intensity of αSMA staining normalized on cell number. **J** Dot plot showing the percentage of αSMA-positive cells. **K**, **L** Dot plots showing *Col1a1*, and *Col1a1/Col3a1* ratio, respectively, measured by realtime qPCR relative gene expression, plotted as fold change normalized on the WT NT samples. For all dot plots, mean ± S.E.M. was indicated. N≥3; * *p* < 0.05; **p < 0.01; ***p < 0.001; ****p < 0.0001.

To clarify the functions of CFs in Charme^KO^ hearts, primary cultures of CFs were established from 5-week-old WT and Charme^KO^ cardiac tissues and subjected to RNA-sequencing analysis (Supplementary Table 2). Of note, WT cultured CFs were representative of the native stromal cells, as shown by the similar percentage of the CD45-/CD309-(collectively lin-)/Sca1+ cells in the established cultures (Supplementary Fig. 2A), as well as by the significant correlation between the two transcriptomes (Supplementary Fig. 2B). The top 300 expressed gene lists of native or cultured CFs were used for GO enrichment analysis which revealed a substantial overlap of the significantly enriched categories (Supplementary Fig. 2C). GO analysis of DEGs in Charme^KO^ versus WT cultured CFs also identified terms related to ECM, mirroring the results observed in native CFs (Supplementary Fig. 2D–F). Cultured Charme^KO^ CFs showed reduced expression of *Col1a1* as compared to WT, while *Col3a1* expression was not modulated (Fig. 2A, B), confirming the expression profile and protein ratio observed in the freshly isolated cells and tissue, respectively. This pattern of gene expression indicates that the altered collagen composition observed in the heart is due to cell-intrinsic defects in the expression of collagen and other ECM-related genes together with a reduced number of CFs in vivo.

The ability of Charme^KO^ CFs to remodel the ECM was then tested in a matrix digestion assay where cells were seeded within a collagen plug to monitor their resorption capacity. The measurement of the area of the collagen matrix showed that Charme^KO^ CFs had reduced matrix digestion capacity compared to WT (Fig. 2C, D). Consistently, flow cytometry analysis of cultured Charme^KO^ CFs showed a significantly reduced proportion of activated lin-/Sca1 + /CD90+ fibroblasts [25] in comparison to WT cells (Fig. 2E, F). Moreover, lower levels of the gene *Thy1*, encoding the CD90 marker, were also observed in the Charme^KO^ CFs by real-time qPCR (RT-qPCR) (Fig. 2G). Overall, these data hint at an impairment of Charme^KO^ CFs capability to become fully activated fibroblasts.

To pursue this hypothesis, Charme^KO^ CFs were subjected to TGF-β1 treatment, which is a known inducer of CF activation [26]. Immunofluorescence staining, performed upon TGF-β1 stimulation, showed that Charme^KO^ CFs had a significantly lower αSMA fluorescence intensity per cell number and a reduced number of αSMA-positive cells, as compared to WT cells (Fig. 2H–J). Moreover, the analysis of RNA-seq data and results by realtime qPCR of untreated cells, focusing solely on the TGFβ-1 pathway gene expression, revealed the downregulation of several components of the signaling machinery in Charme^KO^ CFs, including *Smad2*, *Smad4*, and *TgfbR1* genes (Supplementary Fig. 3A–G). In addition, TGF-β1 treatment increased *Col1a1* gene expression to a lower extent in Charme^KO^ CFs compared to WT (Fig. 2K), with a significantly lower *Col1a1*/*Col3a1* gene expression ratio (Fig. 2L). Hence, Charme^KO^ CFs show impairment of the phenotypic features and functions of mature fibroblasts, and are less responsive to TGF-β1 stimulation for phenotypic conversion into myofibroblasts.

**Fig. 3:**
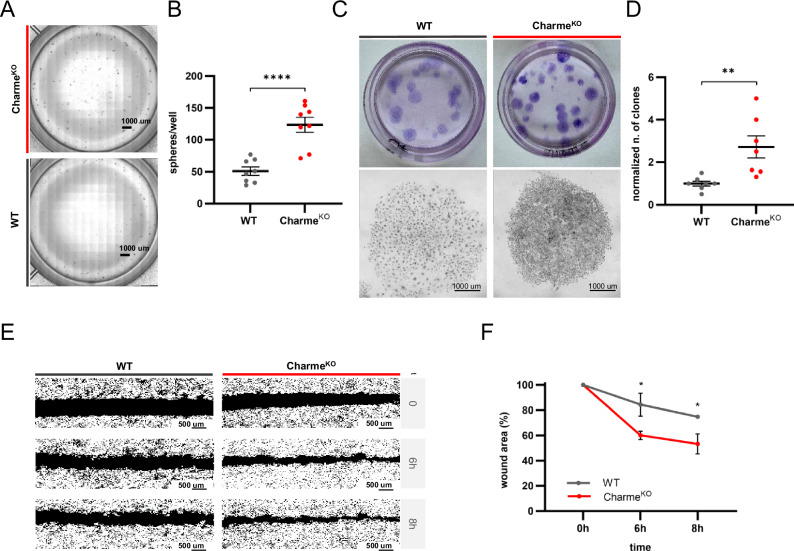
Charme^KO^ cardiac fibroblasts retain features of unactivated mesenchymal cells. **A** Representative phase contrast images of the spheroid formation assay. Images were acquired using Nikon Ti Eclipse microscope with multipoint acquisition at 10X magnification. **B** Dot plot showing the mean number of spheroids per well. **C** Representative images showing the size and number of clones in 60mm Petri dishes (top panels) and single clone cellular density (bottom panels) obtained from clonogenic assays in WT and Charme ^KO^ CFs. **D** Dot plot showing the number of clones per well, normalized on WT. **E** Representative binary masks obtained from ImageJ processing of wound healing assay images at 0, 6 h, and 8 h after scratch. Images were acquired at 10X magnification. **F** Line graph showing the percentage of residual wound area over time. For all dot plots, mean ± S.E.M. was plotted. N≥3; *p < 0.05; **p < 0.01; ****p < 0.0001.

Given the observations of reduced differentiation of Charme^KO^ CFs, multiple assays were performed to assess the retention of features of unactivated fibroblasts, such as motility and self-renewal properties. An assay for spontaneous spheroid formation, a typical feature of mesenchymal cells in culture [27], was performed. Charme^KO^ CFs formed a significantly higher number of spheroids compared to the WT, with comparable size (Fig. 3A, B; Supplementary Fig. 2G). CFs from Charme^KO^ hearts also showed a higher clonogenic ability versus WT cells (Fig. 3C, D), with more evident holoclone-like features of the obtained clones, an index of propensity to self-renewal. Moreover, when subjected to a scratch assay, Charme^KO^ CFs showed higher migration capacities, as compared to WT (Fig. 3E, F). These behaviors observed in Charme^KO^ CF spheroid and clone formation, as well as in cell migration, were not due to a different proliferative capacity, as shown by the similar proliferation rates observed in Charme^KO^ and WT CF cultures at 48 and 72 h from plating (Supplementary Fig. 2H).

These data demonstrate that Charme^KO^ hearts contain a higher proportion of CFs with unactivated phenotypes.

### Charme^KO^ cardiac fibroblasts display impoverished secretome and impaired paracrine functions

**Fig. 4:**
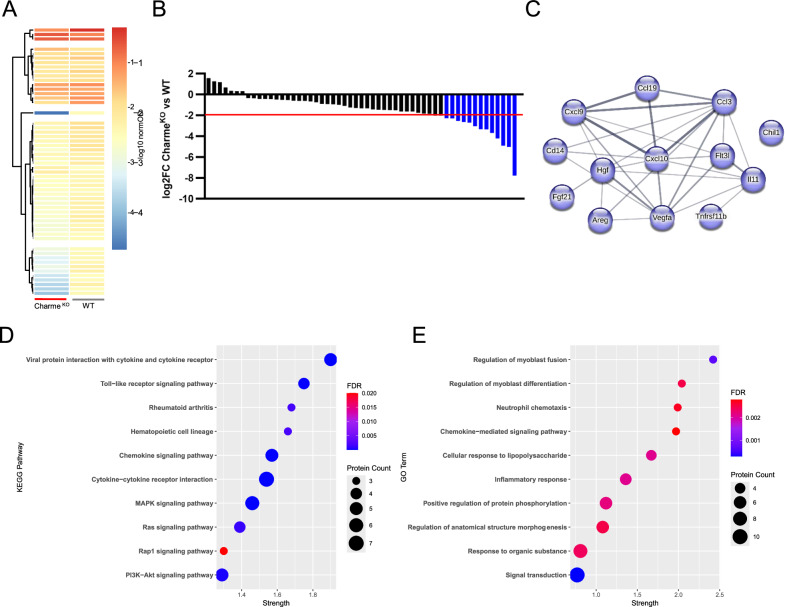
Charme^KO^ cardiac fibroblasts secrete low amounts of cytokines mediating specific signaling pathways. **A** Heatmap showing the clustering of differentially abundant molecules in conditioned media collected from Charme^KO^ CFs versus WT. **B** Bar graph showing the log2 fold-change of the normalized OD obtained in image quantification of the cytokine array used for screening the CF conditioned media. The threshold level for selecting the cytokines to be included in the following analyses is indicated by the red line. **C** Network graph of STRING Functional Network Analysis of selected cytokines, down-regulated (blue) in Charme ^KO^ CFs compared to WT. **D** Bubble graph of the top ten enriched pathway terms retrieved by the KEGG Pathway enrichment analysis of differentially abundant molecules. Numerosity (protein count) and false discovery rate (FDR) of the observed enrichment are reported. **E** Bubble graph of the top ten enriched Gene Ontology terms retrieved by the GO analysis of differentially abundant molecules. Numerosity (protein count) and false discovery rate (FDR) of the observed enrichment are reported.

To better define the features of CFs isolated from Charme^KO^ hearts, we investigated whether their paracrine signaling function was also affected. Since the cardiac stroma exerts some fundamental functions through secreted cytokines [20, 21], conditioned media from WT and Charme^KO^ CFs were screened using an antibody array for the detection of 111 target cytokines. The analysis revealed 78 detectable cytokines in both samples, and 55 of them showed at least a 20% change between Charme^KO^ and WT cells levels (Fig. 4A). Notably, the number of down-regulated cytokines was greater than that of up-regulated ones (Fig. 4B), and they were shortlisted according to a minimum modulation of log2(FC) > | 2| in Charme^KO^ samples as compared to WT. The obtained list of 13 cytokines was used for a STRING network analysis which showed a functional network including 12 of them (Fig. 4C). KEGG pathway analysis returned relevant terms, including “Ras and MAPK“ (respectively, mmu04014 and mmu04010; FDR < 0.005), and the “PI3K/Akt” signaling (mmu04151; FDR < 0.005) pathways (Fig. 4D). These are known to have an important role in cardiac adaptation [28], as well as in response to mechanical and oxidative stress, and to ischemia/reperfusion injury [29, 30]. In line with the previous evidence [7–9], GO analysis also identified significant enrichment in categories related to the regulation of myoblast fusion and differentiation (Fig. 4E).

**Fig. 5:**
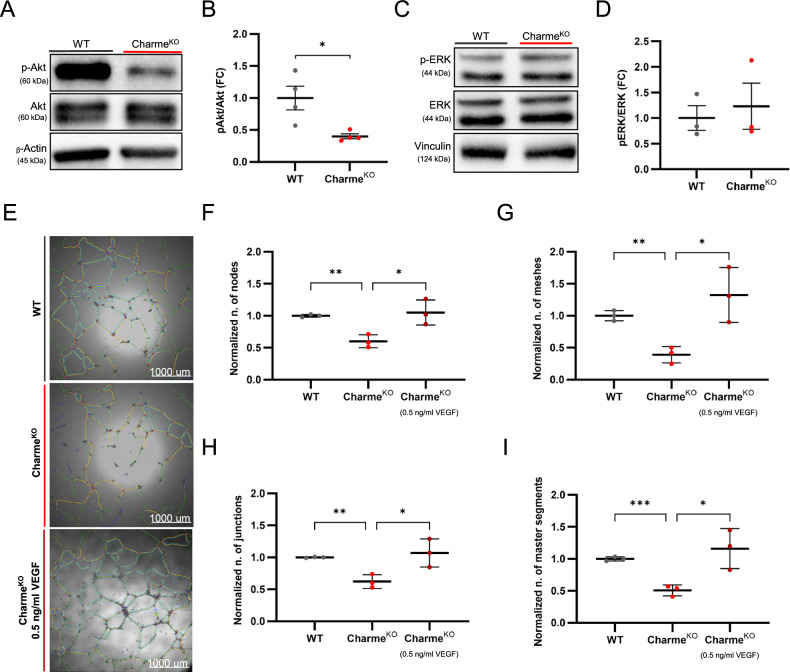
Charme^KO^ cardiac fibroblast conditioned media reduces both AKT phosphorylation in cardiomyocytes and endothelial cell angiogenesis. **A** Representative western blot images showing phosphorylated AKT (P-AKT) and total AKT protein levels in proteins extracted from NRVMs treated with WT or Charme^KO^ CF conditioned media for 1 h. β-actin was used as the loading control. **B** Densitometric analysis of the bands for P-AKT/AKT ratio, showing the Fold Change (FC) normalized over β-actin. **C** Representative western blot images showing phosphorylated ERK (P-ERK) and total ERK protein levels in the proteins extracted from NRVMs treated with WT or Charme^KO^ CF conditioned media for 1 h. Vinculin was used as the loading control. **D** Densitometric analysis of the blots for P-ERK/ERK ratio, showing the Fold Change (FC) normalized over Vinculin. **E** Representative bright field images of tube formation assay with the superimposition of the network masks created by the automated analysis by ImageJ. **F–I** Dot plots showing the number of nodes, meshes, junctions, and master segments respectively, in the tube formation assay, normalized on WT. N≥3; *p < 0.05; **p < 0.01; ***p < 0.001.

Given this notable alteration, the impact of Charme^KO^ CFs paracrine signaling on other cardiac cell types was investigated. Neonatal rat ventricular myocytes were exposed to CF-conditioned media to explore its effect on the signaling pathways activated in cardiomyocytes through paracrine action. Coherently with the KEGG prediction and in line with the impoverishment of secreted cytokines observed in the Charme^KO^ CF secretome, lower levels of phosphorylated AKT were detected by WB in cardiomyocytes treated with Charme^KO^ CF-conditioned media, compared to WT (Fig. 5A, B). In contrast, ERK phosphorylation levels did not show relevant differences (Fig. 5C, D). The analysis of the canonical signaling pathways acting downstream of AKT in cardiomyocytes showed no significant modulation between treatments with Charme^KO^ or WT CF-conditioned media. This likely reflects the absence, in our experimental setting, of a specific stimulus required to trigger a cell survival response, and the activation of the downstream mediators and effectors.

Homeostatic and cardioprotective effects of CFs exerted through paracrine signaling, also include the promotion of angiogenesis [31]. Notably, vascular endothelial growth factor A (VEGFA), which is the main mediator of endothelial cell activation and function, was included among the downregulated cytokines in the Charme^KO^ secretome. The ability of Charme^KO^ CFs to sustain capillary-like structure formation was assessed in a tube-forming assay with human umbilical vein endothelial cells (HUVECs). Conditioned media of Charme^KO^ CFs was significantly less effective in sustaining tube formation compared to WT cells, as assessed by automatic image analysis of nodes, meshes, junctions, and segments, and this phenotype could be rescued by the addition of recombinant VEGF to Charme^KO^ CF conditioned media (Fig. 5E–I). Overall, these data show an impaired paracrine signaling from Charme^KO^ CFs towards both cardiomyocytes and endothelial cells.

### Charme^KO^ cardiac fibroblasts are less supportive of embryonic cardiomyocyte differentiation

**Fig. 6:**
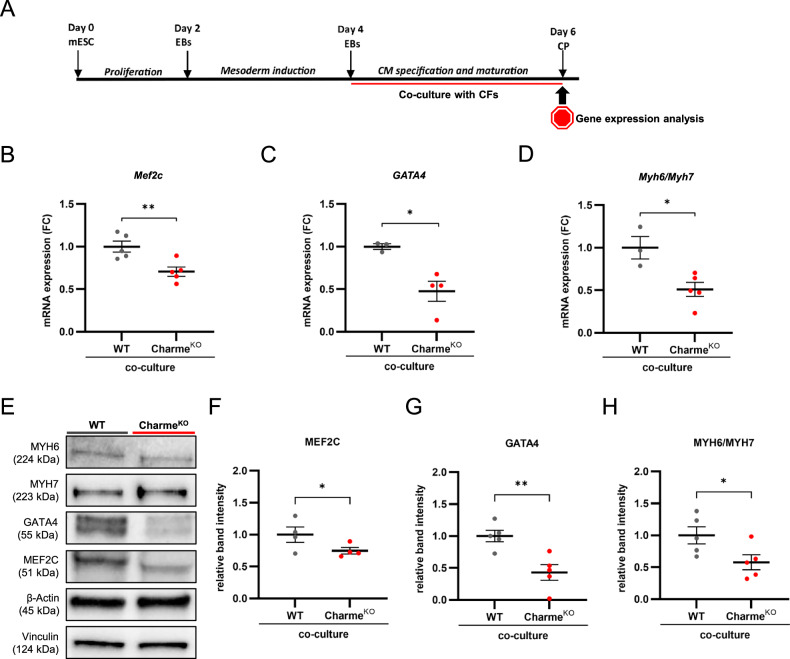
Embryoid bodies co-cultured with Charme^KO^ cardiac fibroblasts show reduced cardiomyocyte maturation. **A** Schematic representation of embryoid body (EB) differentiation and co-culture time course (CM= cardiomyocytes; CP= cardiac progenitors). **B, C** Dot plots showing *Mef2c* and *Gata4* gene expression, respectively, shown as fold change (FC) normalized on the WT samples. **D** Dot plot showing *Myh6/Myh7* gene expression ratio, shown as fold change (FC) normalized on the WT samples. **E** Representative western blot images showing MYH6, MYH7, GATA4, and MEF2C protein levels. β-actin and vinculin were used as loading controls. **F–H** Densitometric analysis of the blots, showing the Fold Change (FC) normalized over the loading controls. N≥3; *p < 0.05; **p < 0.01.

CFs have a well-established role in influencing cardiomyocyte maturation [15]. Given the observed impoverishment of cytokines related to myoblast fusion and differentiation in the Charme^KO^ CF secretome, its paracrine effect on cardiomyocyte differentiation was tested. Specifically, murine embryonic stem cells (mESCs) were used to recapitulate early cardiac development in vitro [32] and test whether Charme^KO^ CFs could be supportive of this process. Cardiomyocyte specification and maturation were induced in E14-Tg2a blastocyst-derived mESCs through the formation of embryoid bodies (EBs), mesoderm induction, and then cardiomyocyte specification, and assessed through the expression of stemness and cardiac specification genes, including *pCharme* (Supplementary Fig. 4A–G). EBs were co-cultured with either WT or Charme^KO^ CFs from day four of differentiation, allowing paracrine communication while avoiding cell-to-cell contact and cross-contamination between the two cell types (Fig. 6A). Markers of cardiomyocyte commitment and maturation were then assessed on day 6 at both mRNA and protein levels [32]. EBs co-cultured with Charme^KO^ CFs displayed significantly lower expression of myocyte enhancer factor 2 C (*Mef2c*) and GATA binding protein 4 (*Gata4*) (Fig. 6B, C), two key transcription factors necessary for the correct expression of muscle and cardiac-specific genes, including myosin heavy chain 6 (*Myh6*), a typical marker of mature adult cardiomyocytes [33, 34]. The *Myh6/Myh7* ratio is widely used to measure cardiomyocyte maturation [35], and EBs co-cultured with Charme^KO^ CFs showed a significantly lower *Myh6/Myh7* expression ratio compared to EBs co-cultured with WT cells (Fig. 6D, Supplementary Fig. 4H, I). Protein levels of the examined genes were further investigated, confirming the significant reduction of MEF2C and GATA4 expression, as well as the reduced MYH6/MYH7 protein ratio (Fig. 6E–H; Supplementary Fig. 4J, K).

The collected evidence shows a defective role of the Charme^KO^ CFs in supporting cardiomyocyte maturation in vitro.

## Discussion

*Charme* is a murine lncRNA, with restricted expression in skeletal and cardiac muscle, involved in the myogenic process [7–9]. In mice, *Charme* knock-out causes a prominent cardiac phenotype characterized by cardiomyocyte hyperplasia and notable alteration of the tissue structure [7]. Its function has been deeply investigated in cardiomyocytes [8, 9], while the effect of the lncRNA ablation on the phenotype and function of non-myocyte cells in the Charme^KO^ heart has not been investigated. The cardiac stroma is a key constituent of the tissue microenvironment,where it plays pivotal roles in tissue development, homeostasis, and pathological conditions. Cardiac stroma acts by modulating cardiomyocyte proliferation and maturation during embryonic development [15], and by maintaining tissue homeostasis in the adult life, through ECM remodeling and paracrine communication with other cell types [20, 36].

Charme^KO^ cardiac tissue displayed an altered ECM composition, with reduced collagen I protein content and Col1/Col3 protein ratio associated with a lower density of resident CFs. Consistently, the analyses on native CFs isolated from Charme^KO^ hearts revealed a peculiar transcriptome, impoverished of genes involved in ECM synthesis and organization, particularly concerning collagen type I. Cultured Charme^KO^ CFs mirrored the reduced expression of genes involved in ECM and collagen organization, and showed reduced matrix digestion ability, indicating altered ECM remodeling and resorption capacity as well.

Cultured Charme^KO^ CFs were depleted of the lin-/Sca1 + /CD90+ population, associated with an activated and pro-fibrotic phenotype in fibroblast maturation [25]. Coherently, Charme^KO^ CFs were less responsive to differentiation into myofibroblasts triggered by TGF-β1. The data collected suggest an impaired maturation of Charme^KO^ CFs into collagen-producing fibroblasts, both in homeostatic and activated conditions. On the other hand, Charme^KO^ CFs showed enhanced mesenchymal phenotype, such as increased spontaneous spheroid growth and cell migration, as well as enhanced clonogenic ability, all features typical of mesenchymal cells in vitro [27], overall resembling an early activation state accompanied by impaired commitment to myofibrogenesis [22].

Besides ECM synthesis and myofibroblast specification, another main function of CFs lays in the communication with other cell types. In this direction, Charme^KO^ CFs revealed a paracrine profile depleted of several cardioprotective molecules involved in cardiomyocyte adaptation and survival pathways. Cytokines involved in the PI3K/Akt signaling pathway were among the most significantly affected by Charme depletion, and this pathway resulted less active in primary cardiomyocytes exposed to Charme^KO^ CF-conditioned medium in vitro. The role of the PI3K/Akt signaling pathway in the heart is well established, since it regulates several cardioprotective processes. Upon injury, the activation of PI3K/Akt signaling pathway promotes cell survival [37, 38] by reducing oxidative stress [39] and inflammation [40], decreasing cardiomyocyte apoptosis [41], and excessive autophagy [42]. The reduced cardioprotective paracrine function of CFs upon Charme depletion was also supported by the observed decrease in angiogenesis, which is consistent with the reduced levels of the pro-angiogenic factor VEGFA in their secretome and its rescue effect on endothelial tube formation.

Altogether, these findings highlight how depletion of Charme in the heart not only affects cardiomyocytes, as previously described [7–9], but also significantly alters the phenotype and function of resident CFs. We speculate that impaired homeostatic function and responsiveness to activation stimuli may potentially affect the ability of Charme^KO^ CFs to face cardiac aging and/or injury, potentially contributing to the heart remodeling observed in the adult Charme^KO^ mice, the development of cardiac dysfunction in the long term [9].

Moreover, CF function has been proven crucial to the determination of correct tissue development, and specifically cardiomyocyte maturation [43]. To this end, the switch from neonatal to adult state of CF is pivotal. The release of specific molecules as well as ECM proteins by CFs drive cardiomyocyte cell cycle exit and promote their structural and functional maturation, mainly through ECM-receptor interaction, focal adhesion, and chemokine signaling [15]. In line with these evidences, the secretome of Charme^KO^ CFs displayed a significant reduction in the levels of cytokines involved in the regulation of myoblast differentiation and fusion, and mediated significant impairment of cardiomyocyte maturation in an in vitro model of cardiac differentiation from mouse embryonic stem cells. These results suggested a direct involvement of CFs in the alterations of the Charme^KO^ cardiomyocyte compartment through paracrine signaling.

Notably, the effect of *Charme* depletion can still be observed in adult CFs despite the lncRNA is not expressed in this cell type. The molecular mechanism whereby *Charme lncRNA* mediates cellular responses beyond its expression remains to be investigated, and possibly lies in the impairment of the paracrine signaling from cardiomyocytes to fibroblasts occurring during heart development, when *Charme* is indeed expressed in cardiomyocytes. Charme knockout directly drives the cardiomyocyte hyperplasia and reduced maturation observed in vivo by controlling myogenic gene expression during embryogenesis [9]. Recent data described signaling occurring from cardiomyocytes to CFs during development, mediated by TGF-β, which promotes early differentiation of CFs to co-evolve the ECM environment necessary for full maturation of functional cardiomyocytes [44]. The effect of TGF-β depletion in vivo in cardiomyocytes during development are still evident in adult CFs, suggesting that the impaired maturation in embryonic life can indeed persist in a dysfunctional phenotype in adulthood. This is to some extent similar to our observations in CFs isolated from adult Charme^KO^ mice, where we hypothesize an impaired paracrine signaling from Charme^KO^ cardiomyocytes to CFs, preventing their full maturation.

Therefore, we speculate that the altered cardiomyocyte maturation, in which Charme plays a direct role, might affect the microenvironment since embryogenesis, mediating a persistently altered intercellular crosstalk with developing CFs, which changes them irreversibly throughout adulthood.

In conclusion our observations point to the presence of an altered intercellular signaling occurring in Charme^KO^ hearts, possibly established by Charme expressing cardiomyocytes during development, which impairs CFs. As *Charme* lncRNA is also expressed in the human heart [45, 46], further investigation will be required in order to identify the pathways affected by cell-cell interaction and promoting cardiomyocyte maturation, the microenvironment homeostasis, and possibly their involvement in different cardiac diseases.

## Materials and Methods

### Cardiac fibroblast isolation and treatment

The Charme^KO^ animals were previously derived through the insertion of a PolyA signal in the Charme locus, as detailed in Ballarino et al. [7]. CFs were either freshly isolated from the cardiac tissue through dissociation and hematopoietic lineage depletion or cultivated through the explant culture protocol. Hearts were explanted from 3 WT and Charme^KO^ C57BL/6 J mouse models at the age of 5 weeks. The isolation of native CFs was performed using a Multi tissue dissociation kit for the dissociation of adult mouse heart from Miltenyi Biotech (Cologne, Germany), following the standard protocol provided (Fig. Supplementary 1A), which allows the isolation of non-cardiomyocyte populations. Briefly, cardiac tissue was harvested and subjected to enzymatic digestion (Multi Tissue Dissociation Kit 2, Miltenyi, #130-110-203) and dissociation through a gentleMACS Dissociator (Miltenyi, #130-093-235), followed by red blood cell lysis. The isolated cells were subjected to immunomagnetic selection for hematopoietic cell lineage depletion, using the Direct Lineage Cell Depletion Kit (Miltenyi, #130-110-470) following the protocol provided.

The explant culture method followed the protocol described [47]: in brief, isolated atrial tissue collected from 6 littermate animals (4–6 weeks of age) was washed with PBS, fragmented with scissors into 1 mm^3^ pieces and digested for 5 min with 0.05% trypsin-EDTA (Lonza, Basel, Switzerland); the resulting pieces were plated in 60 mm Petri dishes previously coated for one hour with 50 ng/ml fibronectin (Corning, Somerville, MA, USA) and cultured for 2 weeks in complete explant media (CEM) [Iscove’s modified Dulbecco’s medium (IMDM) (Sigma-Aldrich, St. Louis, MO, USA) supplemented with 20% FBS (Sigma-Aldrich), 1% penicillin- streptomycin (Sigma-Aldrich), 1% L-glutamine (Lonza), and 0.1 mM 2-mercaptoethanol (Thermo Fisher Scientific, Waltham, MA, USA)]. After 2 weeks, outgrowth cells were collected with mild digestion performing sequential washes with Ca2 + -Mg2+ free phosphate-buffered saline (PBS), 0.48 mm Versene (Thermo Fisher Scientific) for 3 min, and 0.05% trypsin–EDTA (Lonza) for 5 min at room temperature under visual control. Harvests were made weekly up to three times. Cells were cultured in CEM, and early-passage cultures were used for all the experiments.

For TGFβ1 treatment, cells were plated on fibronectin (Corning) at a cell density of 1.8*10^4^ cells/cm^2^ and treated with 10 ng/ml Mouse Transforming Growth Factor β1 (mTGF-β1) (Cell Signaling Technology, Danvers, MA, USA) in CEM for 72 h. The experiment was performed on at least 3 independent primary cell lines established as described above.

### Murine embryonic stem cell differentiation and co-culture

E14-Tg2a mESCs were a kind gift from Dr D.Alfano, and were routinely tested for mycoplasma. Cells were cultured without feeders and maintained undifferentiated on gelatin-coated dishes in GMEM (Sigma-Aldrich; Cat#103 G5154) supplemented with 10^3^ U/ml ESGRO LIF (Millipore, Burlington, MA, USA; Cat# ESG1107), 15% fetal bovine serum (ES Screened Fetal Bovine Serum, US Euroclone, Milan, Italy; Cat# CHA30070L), 0.1 mM non-essential amino acids (Gibco, Cat# 11140-035), 0.1 mM 2-mercaptoethanol (Gibco, Cat# 31350-010), 0.1 mM L-glutamine (Gibco, Cat# 25030081), 0.1 mM Penicillin/Streptomycin (Gibco, Cat# 10378016), and 0.1 mM sodium pyruvate (Gibco, Cat# 11360-070). Cells were passaged every 2–3 days using 0.25% Trypsin-EDTA (1X) (Gibco, Cat# 25200056) as the dissociation buffer. For differentiation, E14-Tg2a mESCs were dissociated with Trypsin-EDTA and cultured in ultra-low attachment plates (Corning) at 1*10^5^ cells/ml in serum-free media: 75% Iscove’s modified Dulbecco’s media (Cellgro, NE, Lincoln, USA; Cat#15-016-CV) and 25% HAM F12 media (Cellgro; #10-080-CV), supplemented with N2 (Gibco #17502048) and B27 (Gibco #12587010) supplements, penicillin/streptomycin (Gibco #10378016), 0.05% BSA (Sigma-Aldrich; Cat#. A9576), L-glutamine (Gibco #25030081), 5 mg/ml ascorbic acid (Sigma-Aldrich; A4544) and 4.5*10^-4 ^M monothioglycerol (Sigma-Aldrich; M-6145). After 48 h in culture, EBs were resuspended in serum-free differentiation media with the addition of 1 ng/ml human activin A (R&D Systems, Minneapolis, MN, USA; Cat#.338-AC), and 1 ng/ml human BMP4 (R&D Systems Cat# 314-BP). The 4-day-old EBs were resuspended in serum-free differentiation media supplemented with 5 mg/ml ascorbic acid (Sigma-Aldrich; A4544) and 4.5*10^-4 ^M monothioglycerol (Sigma-Aldrich; M-6145) for 48 h.

For co-culture experiments, 4-day-old EBs were cultured in serum-free media with 5 mg/ml ascorbic acid (Sigma-Aldrich; A4544) and 4.5*10^-4 ^M monothioglycerol (Sigma-Aldrich; M-6145) in multi-12-well cell culture inserts in the presence of adherent WT and Charme^KO^ CFs seeded at a cell density of 1.6*10^4^ cells/cm^2^, for 48 h. The conditioned media were collected from three independent primary CF cell lines established as described above.

EBs were collected every 2 days. QIAzol lysis reagent (QIAGEN; Cat. No.79306) was added for further RNA extraction, while Pierce RIPA Buffer (Thermo Fisher Scientific; Ref: 89900) with protease and phosphatase inhibitors was used for protein extraction.

### Western blot

Hearts were explanted from WT and Charme^KO^ mice (5 weeks of age) and proteins were isolated from ventricle tissue. Protein extracts were made from ventricle tissues and cell cultures using Pierce RIPA Buffer with protease and phosphatase inhibitors and stored at −80 °C until analysis. Protein lysates were mixed with Laemmli 4× buffer with 5% β-mercaptoethanol and boiled at 100 °C for 5 min, loaded on a sodium dodecyl sulfate 15% polyacrylamide gel for electrophoresis (SDS-PAGE), then transferred to PVDF membranes (Sigma-Aldrich). Membranes were blocked with 3% BSA for 1 h at room temperature, and then incubated with primary antibodies against α/β-Tubulin (Cell Signaling Technology, Danvers, MA, USA), Collagen-I-alpha1 (COL1A1, Santa-Cruz Biotechnology, Dallas, TX, USA), Collagen-III-alpha1 (COL3A1, Santa-Cruz Biotechnology), Vinculin (Cell Signaling Technology), pAkt (Cell Signaling Technology), Akt (Cell Signaling Technology), pERK (Cell Signaling Technology), ERK (Cell Signaling Technology), MYH6 (Proteintech, Manchester, UK), MYH7 (Proteintech), GATA4 (Cell Signaling Technology), MEF2C (Abcam, Cambridge, UK), β-Actin (Cell Signaling Technology) at 4 °C overnight with gentle agitation. Membranes were washed with TBS-0.01% Tween and incubated with appropriate horseradish peroxidase (HRP)-conjugated secondary antibodies (Cell Signaling Technology) for 1 h at room temperature with gentle shaking. Chemiluminescent detection was performed after incubation with Clarity western ECL substrate (Bio-Rad, Hercules, CA, USA), following the manufacturer’s instructions using ChemiDoc XRS+ Imager (Bio-Rad), and analyzed using ImageLab software (Bio-Rad). Each band density was adjusted for background signal and normalized to the loading control. Uncropped images of all the western blots are available in the supplemental material (Supplementary Western Blot file). All antibodies used are listed in Supplemental Table 4.

### Total RNA extraction

Total RNA of native stroma, CFs, and EBs was extracted starting from 1*10^5^ cells using column-based kits (miRNeasy Micro kit, QIAGEN), according to the manufacturer’s instructions. Total RNA of cardiac tissue was extracted from heart tissue using column-based kits (miRNeasy Mini kit, QIAGEN), according to the manufacturer’s instructions. Briefly, for both, a first phase of cell lysis was followed by precipitation in ethanol, RNA binding to the column and several washes to collect total RNA in 14 μl and 30 μl of RNase-free water respectively, that was then quantified on a NANODROP instrument (Thermo Scientific) at 260–280 nm and stored at −80 °C.

### Reverse transcription and Real-time PCR

For reverse transcription, the High-Capacity cDNA Reverse Transcription Kit (Applied Biosystems, Waltham, MA, USA) was used, following the manufacturer instructions. Real time PCR was performed using the SYBR Green Master Mix (Applied Biosystems) on a 7900HT Fast Real-Time PCR System (Applied Biosystems). cDNA from reverse transcription was diluted to the concentration of 10 ng/μl and 1 μl was used for amplification. The final primers concentration was 200 nM; primers efficiency has been previously tested, and their specificity was confirmed through the analysis of melting curves. GAPDH and HPRT were used as endogenous control genes. The relative ratio versus endogenous controls was calculated using the comparative Ct method (2 − ΔCt). (Supplementary Table 3).

### RNA-sequencing

RNA was extracted from three native and three cultured CF (obtained as described above) samples and used for mRNA sequencing performed on Illumina platform. Q30-filtered raw reads were obtained from Illumina BaseSpace Reads and aligned to GMCm39 assembly using STAR aligner software [48]. Gene loci fragment quantification was performed on Ensemble (release 87) gene annotation gtf using STAR –quantMode GeneCounts parameter. The gtf file was edited adding CHARME gene genomic coordinates. Read counts of “reverse” configuration files were combined into a count matrix file, that was given as input to DESeq2 [49] R package for normalization and differential expression analysis, after removing genes with less than ten counts in at least two samples. Adjusted p-value cutoff for selecting significant DEGs was set to 0.05 unless otherwise specified. Heatmap of DEGs was generated using pheatmap R package (Pheatmap: pretty heatmaps. R Kolde. R package version 1 [2], 726) from normalized scaled data.

Volcano plots were generated using Enhanced Volcano R package (https://bioconductor.org/packages/devel/bioc/vignettes/EnhancedVolcano/inst/doc/EnhancedVolcano.html). Enrichment analyses on GO, KEGG pathways, WikiPathays, and Reactome, were performed on up-regulated and down-regulated genes using EnrichR web server and R package [50, 51].

RNA-sequencing analyses were validated through real-time PCR for the evaluation of the expression of genes selected as the most abundantly expressed in our samples and being the three most significant DEGs. The selected genes for the validation of RNA-sequencing analysis on native cardiac stroma are: collagen-1-alpha-1 (Col1a1), elastin (Eln), and fibronectin 1 (Fn1) (Supplementary Fig. 1I). The selected genes for the validation of RNA-sequencing analysis on cultured CFs are: elastin (Eln), myosin heavy chain 11 (Myh11), and ADAM Metallopeptidase With Thrombospondin Type 1 Motif 15 (Adamts15) (Supplementary Fig. 2F).

### Cardiosphere formation assay

Following explant culture protocol, collected CFs from at least three independently established primary lines were seeded in duplicate 24-well plates previously coated for 1 h with Poly-D-Lysine (Corning) at low density (2,5*10^4^ cells/well) in cardiosphere-growing medium (CGM) [35% complete IMDM (Sigma-Aldrich) and 65%, DMEM–F-12 (Sigma-Aldrich) mix containing 3,5% FBS (Sigma-Aldrich), 2% B27 (Gibco), 0.1 mmol/L 2-mercaptoethanol (Thermo Fisher Scientific), 10 ng/mL epidermal growth factor (EGF; Peprotech, Rocky Hill, NJ, USA), 20 ng/mL fibroblast growth factor (FGF, Peprotech),40 nmol/L cardiotrophin-1 (Peprotech), 40 nmol/L thrombin (Peprotech), antibiotics, and 1% L-glutamine (Lonza)]. After 1 week, cells spontaneously formed 3D spheroids, which were manually counted and measured in diameter using the NIS Elements AR Software on the Eclipse Ti Microscope (Nikon®, Tokyo, Japan).

### Colony forming unit (CFU) assay

The colony-forming unit (CFU) assay was performed to calculate clonogenic efficiency. Early passage non-confluent primary CFs were seeded at low density (5 cells/cm^2^) in CEM medium on fibronectin (Corning)-coated 60 mm plates and incubated for 14 days at 37 °C, 5% CO2. Colonies produced were fixed with 4% paraformaldehyde, then stained with Giemsa (Sigma-Aldrich) for 1 h and counted using an optical microscope. A cluster with at least 30 cells was considered as a colony, corresponding to a CFU. Clonogenic efficiency was then calculated as the number of CFUs/100 plated cells. At least three independent primary CF cell lines established as described above were used for the assay, each performed in technical duplicate.

### Scratch assay

To evaluate cell migration, a scratch assay was performed. A total of 10^5^ CFs per well were plated in 12-well plates coated with fibronectin (Corning) in CEM 10% FBS. The scratch was performed after 24 h, and then the cells were washed with PBS and cultured with CEM 2% FBS for 10 h. To evaluate CF migration capacity, images were captured every 2 h with a Nikon Eclipse Ti fluorescence microscope equipped with a motorized stage and NIS-Elements AR 4.30.02 software (Nikon Corporation, Tokyo, Japan). Images were analyzed using ImageJ software (Windows 64-bit Java 8 version, NIH, USA; available at: https://imagej.net/ij/download.html), by an automatic macro for scratch area measurement, normalized to the initial area at T0. At least 3 independent primary CF cell lines established as described above were used for the assay, each performed in technical duplicate.

### Flow cytometry

CFs immunophenotype was assessed by flow cytometry. Semi- confluent cultures were harvested and stained with CD45-PerCP-cy5.5, Flk1-PE, CD90-FITC, Sca1-APC-cy7 (Biolegend, San Diego, CA, USA) antibodies. In detail, 1*10^5^ CSCs per staining were harvested with Accutase (Sigma-Aldrich), centrifuged for 5 min at 300 rcf and resuspended in 100 μl FACS media (PBS-2%FBS) containing 0,05 μg of each antibody and incubated for 30 min at room temperature in the dark. The samples were then washed with FACS media, centrifuged for 5 min at 300 rcf and resuspended in 400 μl FACS media prior to analysis. Single stain samples were used for compensation purposes. Specifically, compensation beads covalently bound to either mouse or rat/hamster IgG were stained in the same condition used for the samples (100 μl FACS media + 0,05 μg of antibody) for 15 minutes at room temperature in the dark. A mixture of cells and beads was used as an unstained control. All data acquisition was performed on a FACS-Aria II platform (BD Biosciences, Franklin Lakes, NJ, USA) equipped with FACSDiva software, also used to calculate the compensation parameters (BD Biosciences). All flow cytometry data were analyzed with FlowJo software (FlowJo LLC). Standard gating strategy was used to select single cells; CD45-/Flk1- cells (cells negative for hematopoietic and endothelial markers) were selected for the analysis of Sca-1 and CD90 positivity. The analysis was performed on three native CFs and on at least three independent primary CF cell lines established as described above. All antibodies used are listed in Supplemental Table 4.

### Matrix digestion assay

CFs were suspended in a clear 3D gel matrix 2 mg/ml Rat Tail Collagen I (Gibco A10483-01) pH=7 and seeded at a concentration of 2*10^4^ cells/µl in complete explant medium (CEM) 20% FBS for 4 days. The area of the collagen matrix was measured at day 0 and 96 h after seeding, using a Nikon Eclipse Ti microscope equipped with a motorized stage and NIS-Elements AR 4.30.02 software. At least three independent primary CF cell lines established as described above were used for the assay, each performed in technical duplicate.

### Conditioned media collection and secretome profiling

Conditioned CEM media were collected after the last 24 h of CF culture, in presence of 0,1% FBS, and then analyzed for the quantification of cytokines on a medium throughput array. Media were centrifuged at 2000 rcf for 5 min to remove cells and debris and then stored at −80 °C until analysis. Culture medium was assayed by the Proteome Profiler Mouse XL Cytokine Array (R&D Systems) to simultaneously detect 111 targets. Briefly, array membranes were blocked with blocking buffer for 1 hour at room temperature, and then a mixture of culture medium and array buffers was added to each membrane and incubated overnight at +4 °C with gentle shaking. Membranes were then washed three times in wash buffer, incubated for 1 h at room temperature in Detection Antibody Cocktail and further washed. After that, membranes were incubated for 30 min at room temperature with HRP-conjugated streptavidin and washed one last time to remove unbound reagents. All incubation steps were performed under agitation on an orbital shaker. Membranes were finally incubated with the supplied Chemi Reagent Mix following the manufacturer’s instructions for luminescent spots detection. Arrays were scanned with ChemiDoc Imaging System (Biorad) and spot signal densities were obtained using ImageLab software (Biorad). The background spot signal was subtracted from each specific spot density data and the obtained values were normalized to the positive control spots for each membrane. A heatmap was generated using the log10 value of the densitometric quantification for each dot, using the R package pheatmap (GNU Project). Euclidean distance was calculated using hclust clustering methods implemented in R software. Functional associations network using the STRING database and KEGG Pathways and GO analysis was carried out using Cytoscape (ELIXIR Core Data Resources).

### Tube-formation assay

Human umbilical vein endothelial cells (HUVECs) were plated in duplicate (2.5 × 10^4^ cells/well) on Matrigel-coated 96-well plates (Growth Factor Reduced Matrigel Matrix Phenol Red Free, BD Biosciences) and cultured for 18 h in the CF-conditioned media previously collected from WT and Charme^KO^ CFs. Recombinant VEGF (Peprotech)was added at 0,5 ng/ml. CM were collected from three independently established CFs primary lines. Endothelial growth media (EGM, Lonza) was used as a positive experimental control. The Angiogenesis Analyzer Plugin of ImageJ Software (NIH) was used on randomly captured images with a 4× objective on a Nikon Eclipse TI inverted microscope (Nikon). Cryopreserved HUVECs from pooled donors were purchased from Lonza (C2519A).

### Immunostaining and fluorescence microscopy analysis

For immunofluorescence, CFs were fixed for 15 min with 4% paraformaldehyde at 4 °C, and permeabilized with 0.1% Triton X-100 (Sigma-Aldrich) in PBS with 1% BSA (Sigma-Aldrich). Nonspecific antibody binding sites were blocked with 10% goat serum (Sigma-Aldrich) in PBS.

Murine hearts were collected from 5-week-old WT and Charme^KO^ mice (3 animals), washed in PBS, and embedded in Tissue-Tek® O.C.T. Compound (Sakura Finetek, Torrance, CA, USA). 7µm-slices were cut and fixed for 15 min with 4% paraformaldehyde. Permeabilization was performed with 0.3% Triton X-100 (Sigma-Aldrich) in PBS with 1% BSA. Nonspecific antibody binding sites were blocked with 2% goat serum (Sigma-Aldrich) and 5% BSA (Sigma-Aldrich) in PBS.

Primary antibodies were incubated overnight at 4 °C according to datasheet instructions. After thorough washing, slides were incubated for 2 h at room temperature with the appropriate Alexa-conjugated secondary antibodies (A11029, A1103, Thermo-Fisher) and HOECHST 33258 nuclear dye (Sigma-Aldrich). Slides were mounted in VECTASHIELD® anti-fade mounting medium (Vector laboratories). Image capture was performed on a Nikon Eclipse Ni microscope equipped with VICO system and NIS-Elements AR 4.30.02 software with a 20X objective (Nikon). Fluorescence intensity was normalized to the number of nuclei per each field. All antibodies used are listed in Supplemental Table 4.

### Rat ventricular myocyte isolation and treatment

Hearts were harvested from ten neonatal littermate rats 1–3 days after delivery, atria were removed, and the ventricles were minced in small pieces in PBS at room temperature. Then, tissue was digested through the commercial kit Neonatal Heart Dissociation Kit, mouse and rat (Miltenyi Biotec; #130-098-373), obtaining a single cell suspension, and cardiomyocytes were negatively selected through an antibody-based chromatographic column (Miltenyi Biotec; # 130-105-420) following the manufacturer instructions. Plates were coated with 1% gelatin (Sigma-Aldrich; G1890), and cardiomyocytes were diluted 10^6^/ml in custom-made ‘seeding medium’ (10% horse serum, 100 µM BrdU). 24 hours later, cells stably adhered and the medium was replaced with a serum-free ‘cardiomyocyte medium’ (CM medium), reconstituted from lyophilized DMEM/F-12 (Sigma-Aldrich; D0547) and enriched with 0.72 g/l glucose (Sigma-Aldrich; # G5400), 0.33 g/l sodium pyruvate (Thermo Fisher Scientific; # BP356), 0.017 g/l ascorbic acid (Gibco; # 13080-23), 2 µl selenite 0.2 M (Sigma-Aldrich; # S5261), 0.004 g/l transferrin (Sigma-Aldrich; # T3309), 2 g/l BSA fraction V (Amresco; # 0332), 3.57 g/l HEPES (Amresco; # 0511), 2.43 g/l sodium bicarbonate (Sigma-Aldrich; # S6014) and 10 ml penicillin-streptomycin (Gibco; 15070063).

For conditioned media treatment, NRVMs were plated in duplicate in 96-well plates at a density of 5*10^4^/cm^2^ in 100 μl CM medium. After 3 days of cell culture, NRVMs were treated for 1 h with conditioned media collected from 3 independent WT and Charme^KO^ CFs primary lines.

### Statistical analysis

Statistical analysis was performed using GraphPad Prism 8 software. All results are presented as mean value ± standard error of the mean. Significance of the difference between two groups was determined by two-sided Student’s t-test. When 3 or more groups were specifically intercompared, the parametric one-way ANOVA test followed by the Bonferroni correction was used. A value of *p* < 0.05 was considered as significant.

No formal statistical methods were used to pre-determine sample size. The number of biological replicates was chosen based on previous studies using similar primary cell models and experimental endpoints, as well as on feasibility and reproducibility considerations. Each experiment was performed using *n* ≥ 3 independent primary cell isolations, with technical replicates included for each condition. This sample size is expected to allow detection of biologically meaningful differences between experimental groups.

## Supplementary information

Supplemental Material

Supplementary Figure 1

Supplementary Figure 2

Supplementary Figure 3

Supplementary Figure 4

Supplemental-WB

Supplemental Table 1

Supplemental Table 2

Supplemental Table 3

Supplemental Table 4

## Data Availability

The datasets generated and analyzed during the current study are available in the Gene Expression Omnibus database at the following links https://www.ncbi.nlm.nih.gov/geo/query/acc.cgi?acc=GSE296379https://www.ncbi.nlm.nih.gov/geo/query/acc.cgi?acc=GSE296380.
