# Supplementary material for: Effect of the cardiac long non-coding RNA Charme depletion on the maturation and paracrine signaling of resident cardiac fibroblasts
Source: Cell Death Dis. 2026 Apr 15;17(1):507. doi: 10.1038/s41419-026-08636-x (PMC13201650; doi:10.1038/s41419-026-08636-x)
Supplement: Supplementary file 1 — Supplemental Material [file 41419_2026_8636_MOESM1_ESM.docx]

**List of Supplemental Material**

- Supplemental Figures
- Supplemental Figure legends
- Supplemental Tables
- Supplemental blot file
- Supplemental Methods
- Dataset access number

**Supplementary Figure 1. Native cardiac fibroblast isolation and transcriptomic characterization in Charme^KO^ and WT samples.**

A) Graphical representation of the experimental protocol used for native CF isolation from murine hearts. B) Gating strategy used for flow cytometry analysis of native cardiac stroma obtained through tissue dissociation and hematopoietic cell depletion. C) Bar-graph showing the quantitative real-time PCR for pCharme expression levels in the cardiac tissue, native stroma, cardiac fibroblasts, and lung fibroblasts, plotted as fold change (FC). D) Bubble graph showing terms from the GO analysis of the top 300 expressed genes in native CFs. Strength of association, adjusted p value and enrichment of each category are reported. E) Principal Component Analysis of RNA-Sequencing from WT and Charme^KO^ native cardiac stromal cells. F) Heatmap with hierarchical clustering showing the sample correlation based on the expression data of WT and Charme^KO^ native CFs. G) Bubble plot showing the GO analysis of the DEGs upregulated in Charme^KO^ vs WT CFs. Numerosity of each category, adjusted p value, and odds ratio of the enrichment terms are reported. H) Heatmap with hierarchical clustering showing the expression data and clustering of all DEGs included in the ECM related terms indicated by GO and Reactome database analyses. I) Boxplot showing normalised RNAseq counts for all the collagen genes significantly downregulated in Charme^KO^ vs WT native CFs. J) Bar-graph showing the quantitative real-time PCR validation of selected genes in RNAs from Charme^KO^ vs WT native CFs. K) Representative confocal microscopy images on heart sections from CharmeKO and WT mice after immunofluorescence staining for collagen III (green) and troponin I (red), counterstained for nuclei (blue). L) Quantification of mean fluorescence intensity for collagen III immunostaining in Charme^KO^ heart sections. For all panels: Quantification was made on three independent biological replicates. *= p<0.05; **=p<0.01; ***=p<0.001.

**Supplementary Figure 2. Cardiac fibroblast isolation and characterization ex vivo.**

A) Dot plot showing flow cytometry analysis of native and cultured cardiac fibroblasts (CFs), revealing similar percentage of the lin-/Sca1+ cell population. B) Dot plot showing the Pearson correlation based on the RNA-seq data between genes expressed in native versus cultured WT CFs. C) Bubble graph showing terms from the GO analysis of the top 300 expressed genes in cultured CFs. Strength of association, adjusted p value, and enrichment of each category are reported. D) Bubble plot showing the GO analysis of the DEGs in Charme^KO^ vs WT cultured CFs. E) Boxplot showing normalised RNA-seq counts for all the genes included in the categories shown in panel D in Charme^KO^ vs WT cultured CFs. F) Bar-graph showing the quantitative real-time PCR validation of selected genes in RNAs from Charme^KO^ vs WT cultured CFs. G) Dot-plot showing the measured mean diameter of spheroids generated from Charme^KO^ and WT cultured CFs. H) Line graph showing normalized absorbance at 490 nm for an MTT viability assay. Quantification was made on three independent biological replicates. Values were normalized to t0. For all panels: Quantification was made on three independent biological replicates. Mean ± S.E.M. was plotted. *= p<0.05; **=p<0.01; ***=p<0.001.

**Supplementary Figure 3. Analysis of the expression of genes involved in the TGFβ signalling pathway in Charme^KO^ CFs.**

A,B) Heatmaps with hierarchical clustering showing the expression of (A) all the genes, and (B) the differentially expressed genes (DEG p<0,1) from RNA-seq data involved in the TGFβ signalling pathway in WT and Charme^KO^ in native CFs. C-G) Dot plots showing the quantitative real-time PCR for the TGFβ-signalling pathway genes Smad2, Smad3, Smad4, Tgfbr1, Tgfbr2 in WT and Charme^KO^ in cultured CFs. Mean ± S.E.M. was plotted. **=p<0.01; ***=p<0.001

**Supplementary Figure 4. Differentiation of murine cardiomyocytes from murine embryonic stem cells (mESCs).**

A) Schematic representation of embryoid bodies (EBs) differentiation time course (CM= cardiomyocytes; CP= cardiac progenitors). B) Line graph showing the mean expression level by realtime qPCR of the stemness genes *Nanog* and *Oct3/4* during the differentiation process. C-G) Line graphs showing the expression by realtime qPCR of the cardiac specification genes *Islet1 (Isl1)*, *Mef2c*, *Troponin I (TnI)*, *Cacna1c*, and *pCharme,* respectively, during the differentiation time course. Relative gene expression of each mRNA is reported as fold change normalized on day 0. H,I) Dot plots showing the quantitative realtime qPCR at day 6 for Myh6 and Myh7 gene expression, respectively. J,K) Densitometric analysis of the western blots for MYH6 and MYH7, respectively, with the band intensity normalised over vinculin. Quantification was made on at least three independent biological replicates. Mean ± S.E.M. was plotted. *= p<0.05.

**Supplemental methods**

## **MTS cell viability assay**

MTS cell viability assay was performed to evaluate cell proliferation of cultured CFs using Cell Titer 96® Aqueous Non-Radioactive Cell Proliferation Assay (MTS) (Promega, Madison, WI, USA). Three independent WT and Charme^KO^ CFs primary lines were plated in triplicate in 96-well plates at a density of 5*10^4^ cells/cm^2^ in 100 μl of CEMmedium. After 48 and 72 hours,viability was measured adding 20 μl of combined MTS/PMS Solution to each well and incubating for 1 hour. The absorbance at 490 nm was recorded using Varioskan™ LUX Multimode Reader (Thermo Fisher Scientific) and normalized on T0 absorbance.

**Dataset access number**

Token for reviewer access to GEO

dataset GSE296379: **onmvioiuhzafxad**

dataset GSE296380: **ylotioiwvlsnhin**
