# Supplementary figures and images for: Effect of the cardiac long non-coding RNA Charme depletion on the maturation and paracrine signaling of resident cardiac fibroblasts
Source: Cell Death Dis. 2026 Apr 15;17(1):507. doi: 10.1038/s41419-026-08636-x (PMC13201650; doi:10.1038/s41419-026-08636-x)

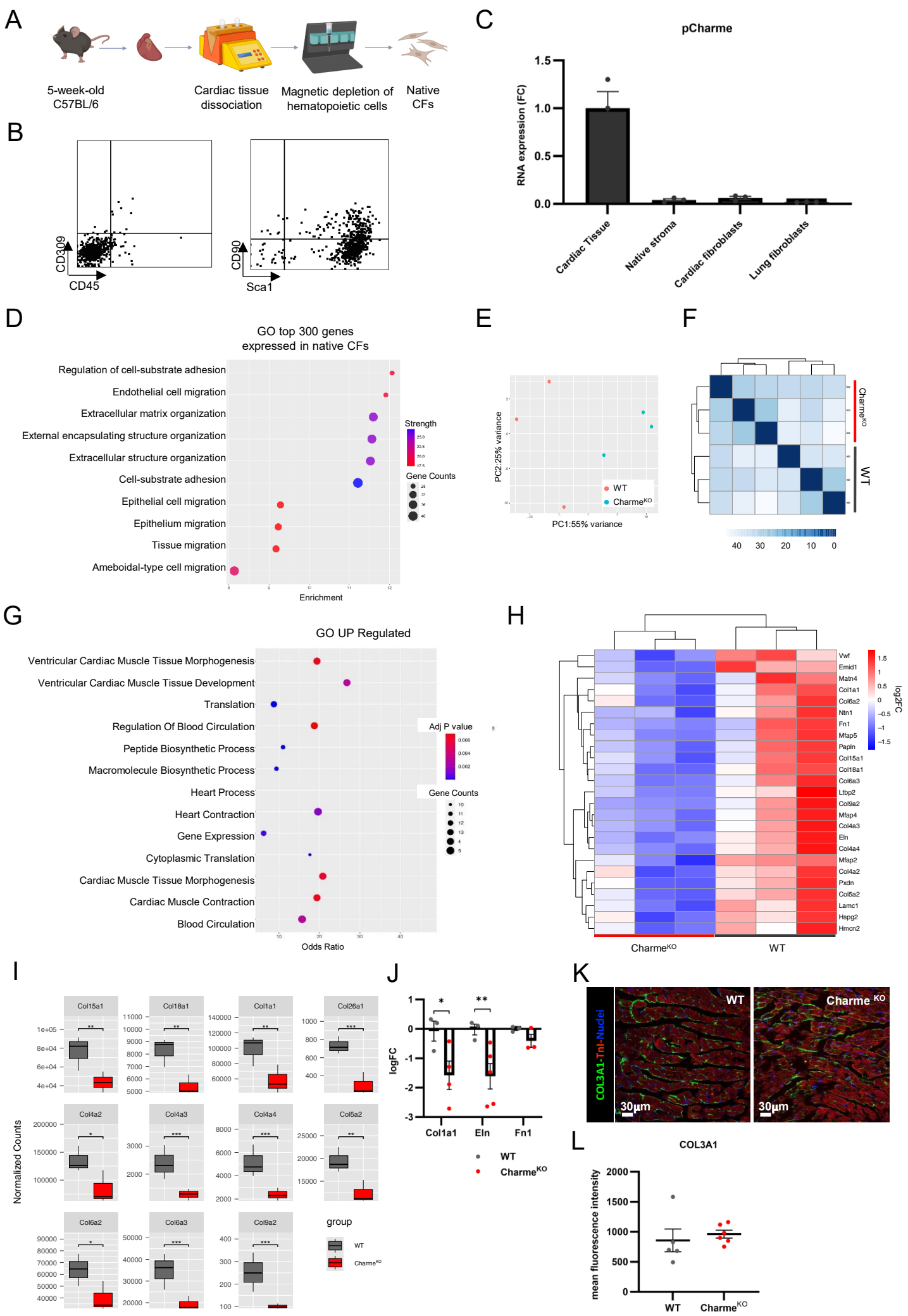

Supplement: Supplementary file 2 — Supplementary Figure 1 [file 41419_2026_8636_MOESM2_ESM.pdf]

A

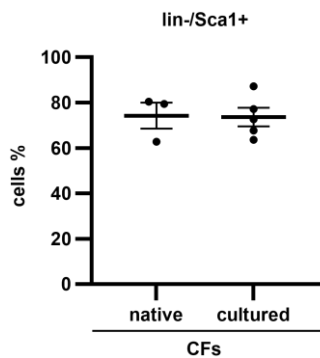

B

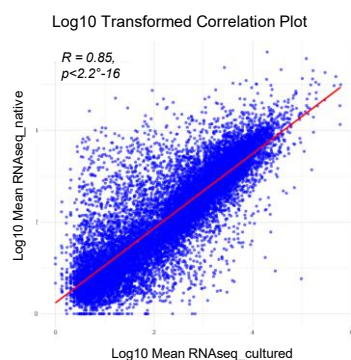

C

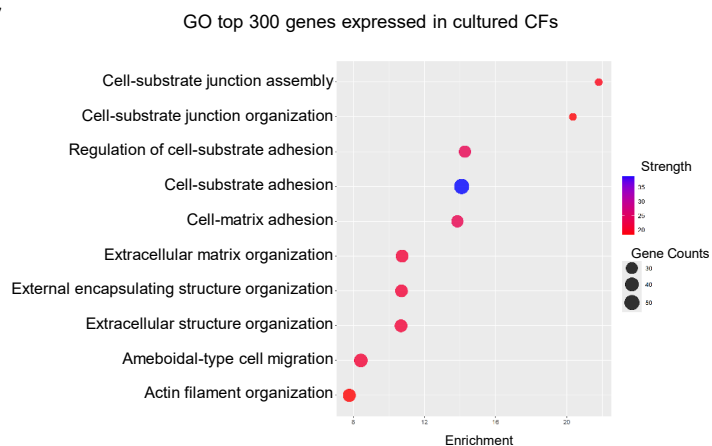

D

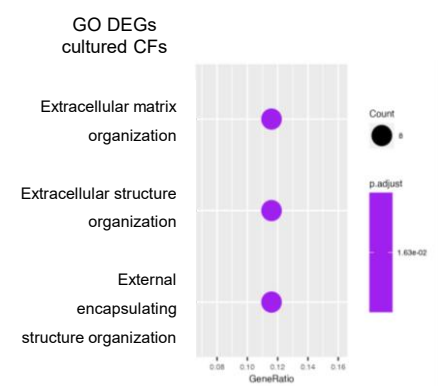

E

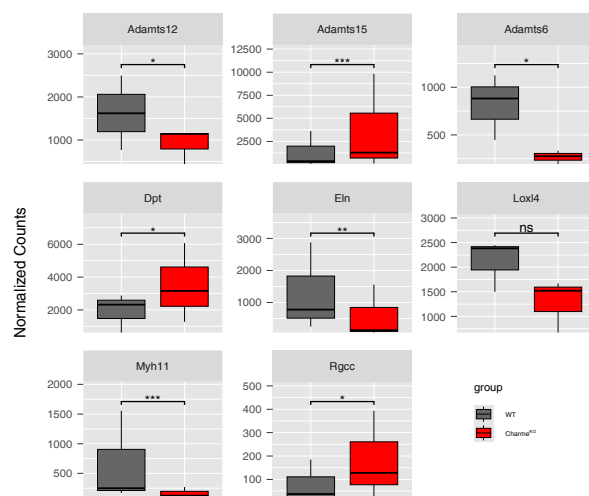

F

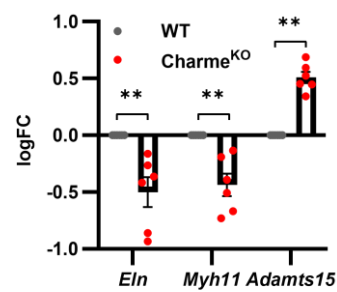

G

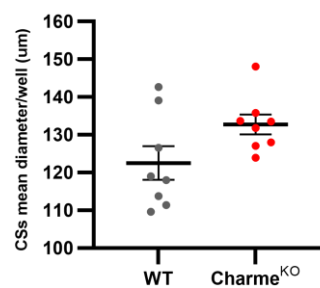

H

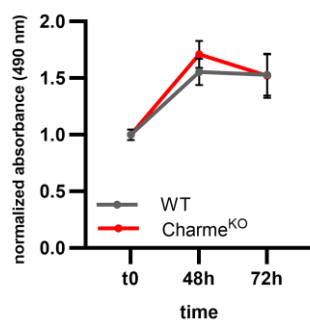

Supplement: Supplementary file 3 — Supplementary Figure 2 [file 41419_2026_8636_MOESM3_ESM.pdf]

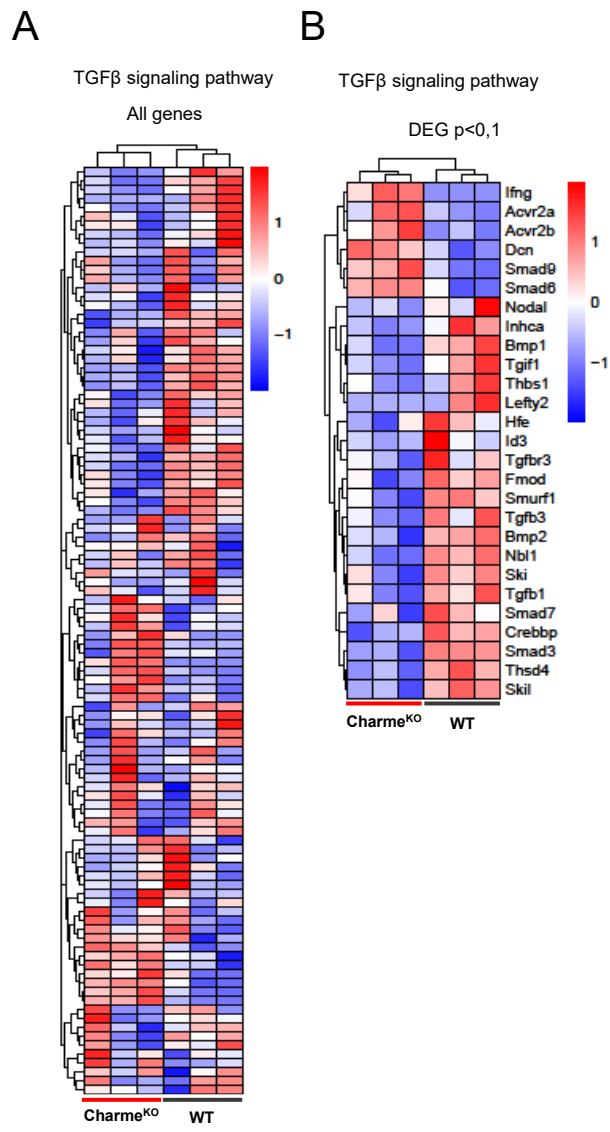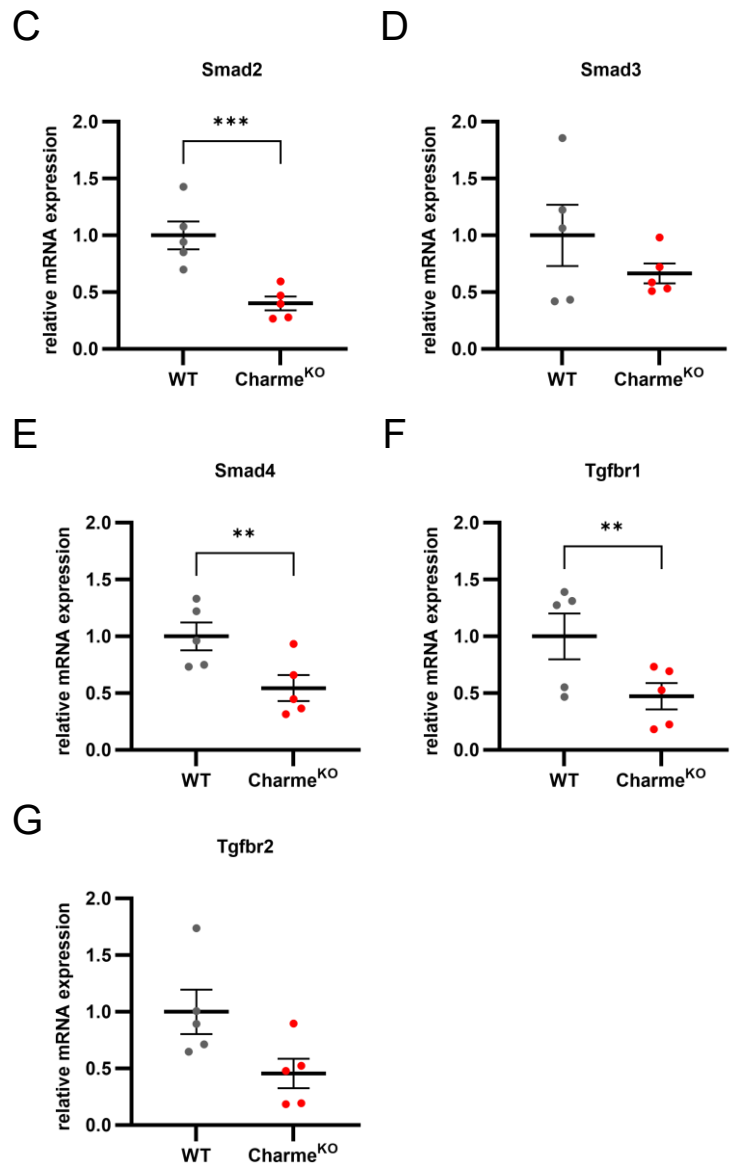

Supplement: Supplementary file 4 — Supplementary Figure 3 [file 41419_2026_8636_MOESM4_ESM.pdf]

A

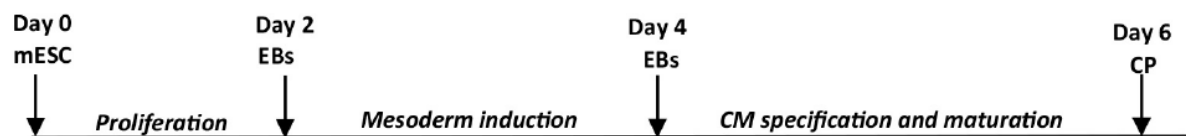

B

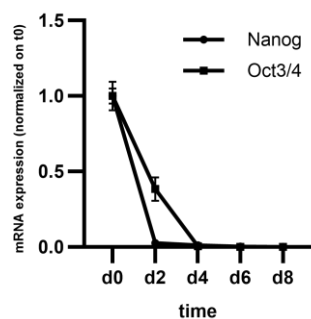

C

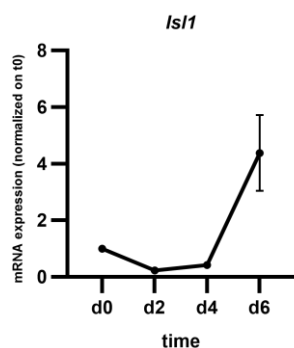

D

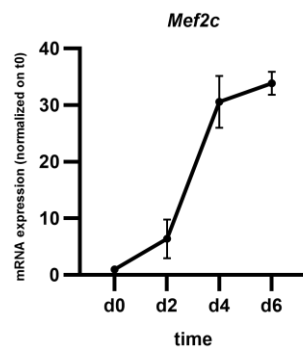

E

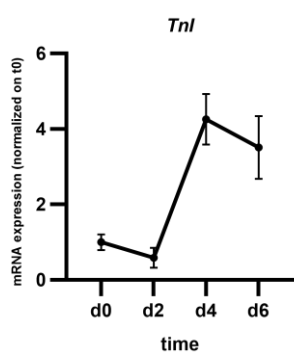

F

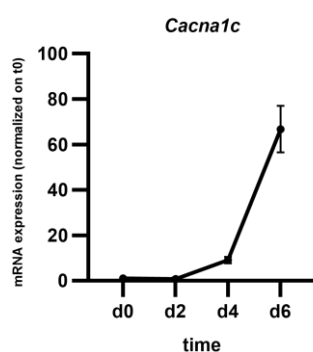

G

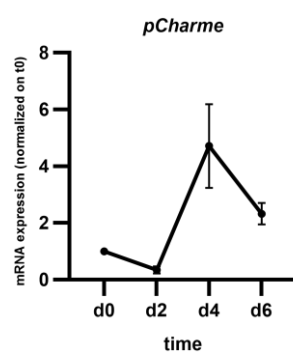

H

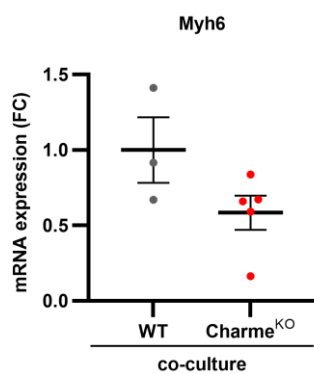

I

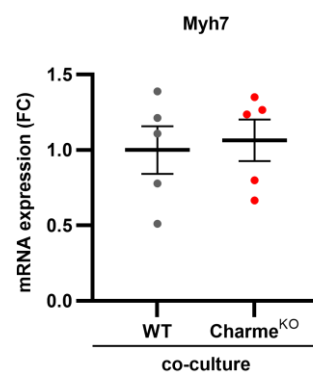

J

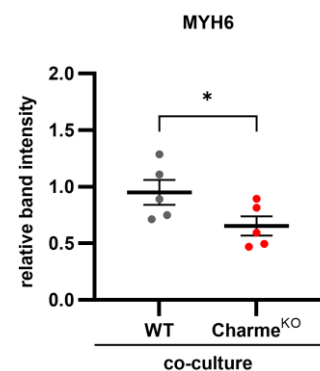

K

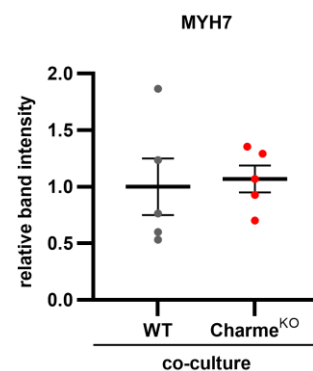

Supplement: Supplementary file 5 — Supplementary Figure 4 [file 41419_2026_8636_MOESM5_ESM.pdf]
