# Supplementary material for: Effect of the cardiac long non-coding RNA Charme depletion on the maturation and paracrine signaling of resident cardiac fibroblasts
Source: Cell Death Dis. 2026 Apr 15;17(1):507. doi: 10.1038/s41419-026-08636-x (PMC13201650; doi:10.1038/s41419-026-08636-x)
Supplement: Supplementary file 6 — Supplemental-WB [file 41419_2026_8636_MOESM6_ESM.pdf]

CARDIAC TISSUE WESTERN BLOT

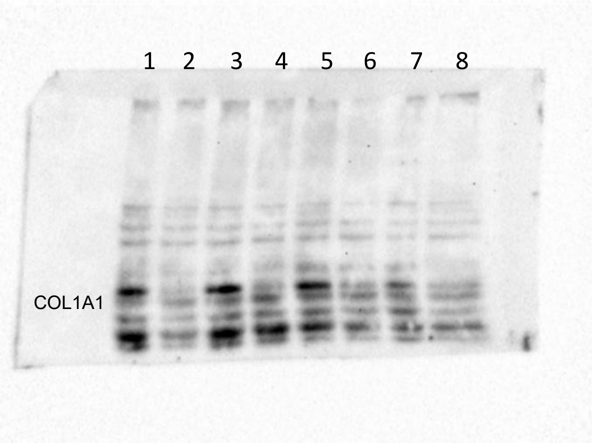

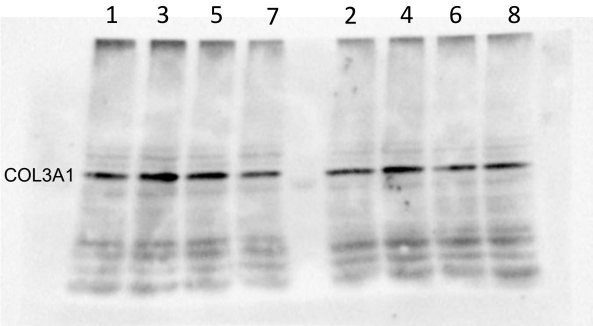

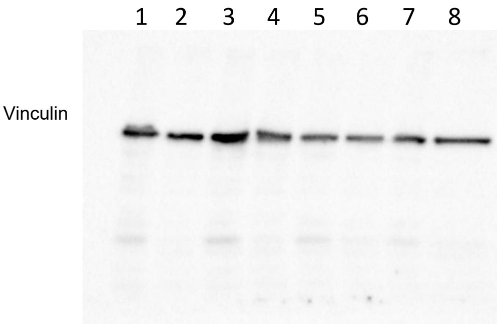

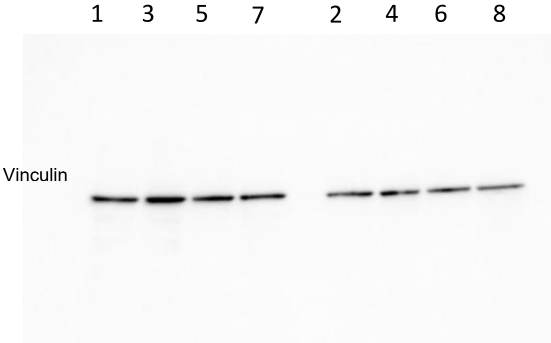

	MOUSE TISSUE SAMPLE
1	WT_#1
2	$CHARME^{KO}$_1
3	WT_#2
4	$CHARME^{KO}$_2
5	WT_#3
6	$CHARME^{KO}$_3
7	WT_#4
8	$CHARME^{KO}$_4

NEONATAL RAT VENTRICULAR MYOCYTE WESTERN BLOT

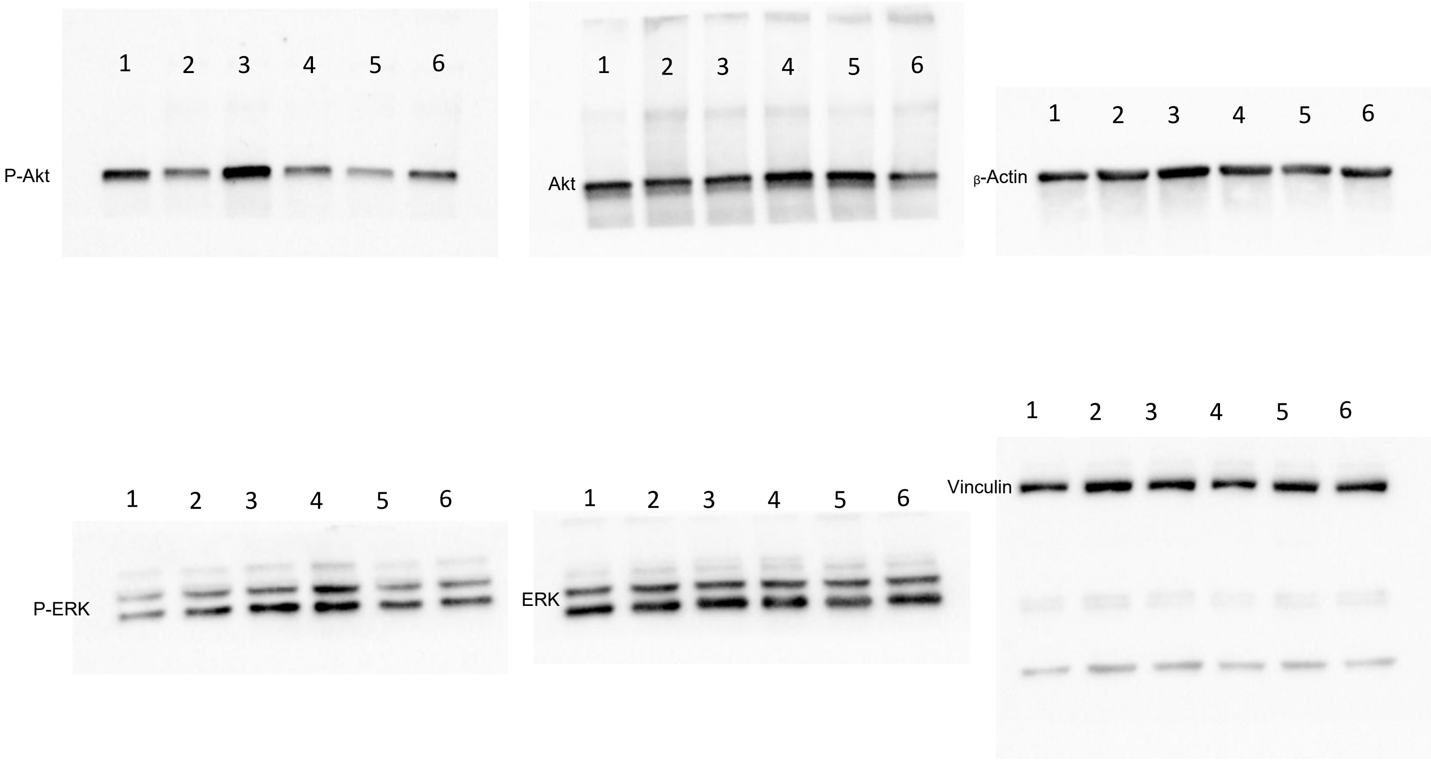

	MOUSE COND. MEDIA TREATMENT
1	WT-CFs_#1
2	CHARMEKO-CFs_#1
3	WT-CFs_#2
4	CHARMEKO-CFs_#2
5	WT-CFs_#3
6	CHARMEKO-CFs_#3

EMBRYOID BODY (DAY 6 OF DIFFERENTIATION) WESTERN BLOT

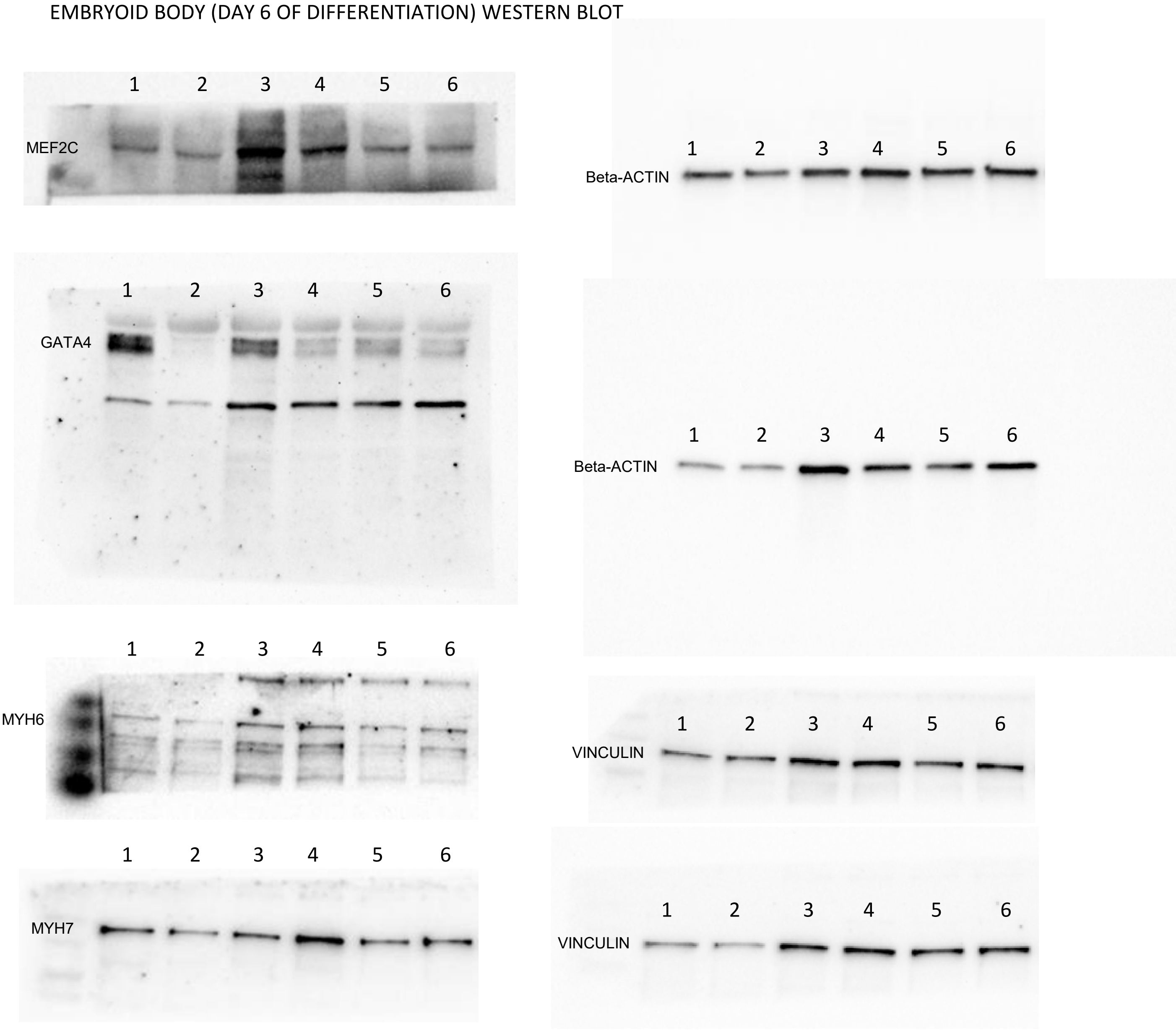

	CARDIAC FIBROBLAST CO-CULTURE
1	WT-CFs_#1
2	CHARME-KO-CFs_#1
3	WT-CFs_#2
4	CHARME-KO-CFs_#2
5	WT-CFs_#3
6	CHARME-KO-CFs_#3

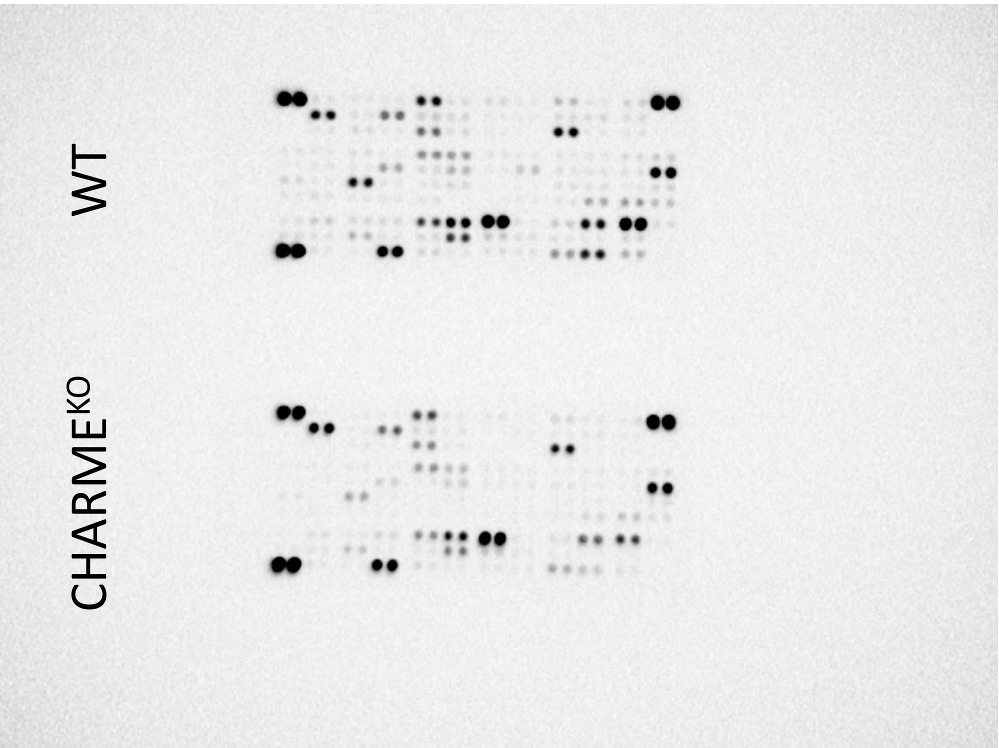

Supplementary file Proteome Profiler
